# Cognitive Processing and EEG Complexity

**DOI:** 10.3390/e28070761

**Published:** 2026-07-03

**Authors:** Antonio J. Ibáñez-Molina, Sergio Iglesias-Parro, M. Carmen Gálvez-Garzón, María Felipa Soriano

**Affiliations:** 1 Department of Psychology, University of Jaén, Campus Las Lagunillas s/n, 23071 Jaén, Spain; 2 St. Agustín University Hospital, 23700 Linares, Jaén, Spain

**Keywords:** EEG complexity, cognitive processing, non-linear analysis

## Abstract

Cognitive neuroscience has addressed the understanding of human brain processes through numerous techniques and psychological paradigms. In general, different types of tasks have been used depending on the specific cognitive operation under study. Since these tasks are usually designed to register responses at the single-trial level, the most common methodological approach to electroencephalography (EEG) is to obtain event-related potentials (ERPs). Crucially, the linear analysis methods associated with ERPs often overlook the intrinsic non-linear and multiscale dynamics of brain activity. Hence, to better characterize brain activity, there is increasing interest in the study of the non-linearity and complexity of EEGs. Given that experiments relating cognitive processing and EEG complexity are still scarce, this work is a narrative review of studies in which non-clinical cognitive processing, such as memory, perception, or attention, is addressed using complexity measures. Here, we focus on EEG metrics derived from the concepts of fractality, information, and randomness across different temporal and spatial scales. We discuss how these measures complement more classical analyses, try to integrate the findings using a predictability–regularity framework, and finally, we point out possible future directions with which to advance current knowledge about the relationship between cognition and EEG complexity.

## 1. Introduction

In contemporary cognitive neuroscience, electroencephalography (EEG) analysis has evolved from a predominantly linear framework toward a non-linear, dynamical perspective on brain function. Early work in this field relied on trial-averaged event-related potentials and band-limited spectral power to relate oscillatory activity to cognitive operations such as memory, attention, and perception [1,2,3,4]. While this approach has enabled significant advances in understanding the association between oscillatory activity and cognitive functions, it has largely treated EEG signals as the output of stationary linear systems, thereby overlooking key non-linear properties of neural dynamics [5,6,7]. In contrast, dynamical systems theory conceives brain activity as emerging from a high-dimensional non-linear system characterized by multiple attractors, metastable transitions, and rich spatiotemporal structure [7,8]. EEG is particularly well-suited to probe these dynamical properties due to its millisecond temporal resolution and its sensitivity to ongoing large-scale spatiotemporal brain dynamics unfolding across multiple temporal scales [9,10].

In this framework, the brain is considered a complex adaptive system whose hierarchical networks give rise to non-linear phenomena with emergent properties. Often underlying this approach is the idea that optimal function occurs in an intermediate regime between rigid order and randomness, balancing stability and flexibility so that the system can reconfigure itself rapidly while maintaining a coherent organization [7,8,11,12].

Given this non-linear nature of brain function, there has been growing interest in the relationship between complexity measures and cognitive functions. Complexity measures quantify the unpredictability of the signal, the temporal structure, and the effective number of independent functional sources contributing to the EEG signal. However, the complexity of mathematical algorithms and the conceptual challenge of interpreting EEG complexity have hindered consistent conclusions about EEG complexity and cognitive functions [5,13,14]. In addition, there are a number of complexity measures, and there is no consensus regarding which measures best capture the temporal and spatial structure of the brain and the synchronization of brain networks. Finally, although early studies showed low brain complexity in aging and certain clinical conditions (the “loss of complexity” hypothesis [10,11]), subsequent studies indicate that the direction of change depends on the specific system, the timescale, and the task demands. Therefore, it cannot be assumed a priori that complexity necessarily decreases with declining cognitive function in aging or disease [12].

While recent reviews have extensively covered the clinical applications of EEG complexity [15] or its methodological foundations [16], there is a lack of a systematic integration focused on how these metrics contribute to our understanding of cognitive functions. This review fills that gap by proposing a functional framework that links signal complexity and different cognitive operations.

This work integrates findings on EEG complexity across different cognitive domains to elucidate the relationship between non-linear brain dynamics and cognitive functions. A literature search was conducted across the PubMed, Web of Science, and Scopus databases, covering publications up to 2026. The search terms included combinations of ‘EEG complexity’, ‘Non-linear dynamics’, ‘Entropy’, ‘Fractal Dimension’, and ‘Lempel–Ziv complexity’, paired with cognitive domains such as ‘perception’, ‘attention’, ‘working memory’, ‘long-term memory’, and ‘executive functions’. Studies were selected based on their relevance to experimental cognitive tasks in healthy populations, while purely clinical or engineering-oriented studies (e.g., BCI optimization without cognitive modeling) were excluded to maintain focus on cognitive functions and neuroscience. First, we will provide a brief summary of complexity metrics, highlighting their differences and particular contributions. Second, we will summarize studies about EEG complexity across cognitive functions. Finally, we will try to integrate results into an explanatory framework.

## 2. Non-Linear EEG Complexity Metrics: Concepts and Computation

As we mentioned before, a number of complexity metrics have been employed within electrophysiological research. Here, we will briefly describe the most important metrics. Following Lau et al. [15], we have categorized them into two groups: metrics that measure the predictability of a system, and metrics that reflect the regularity of a system. Predictability refers to the temporal evolution of the system, whereas regularity refers to the general number of repetitions of patterns in the system’s trajectory.

### 2.1. Predictability

Early complexity metrics tried to capture the predictability of a system by evaluating the temporal correlation in the time series. These approaches aim to assess the extent to which future states can be inferred from past dynamics, typically by characterizing the deterministic structure of the signal.

Within this framework, several classical measures have been proposed. The correlation dimension (D2) estimates the dimensionality of the underlying dynamical system, providing an index of how many variables are effectively required to describe its behavior [17]. Complementarily, Lyapunov exponents (LEs) quantify the rate of divergence of nearby trajectories in the phase space, reflecting the sensitivity to initial conditions and the subsequent presence of chaotic dynamics [18]. The Kolmogorov–Sinai entropy (KSE) provides a measure of the rate of information production in the system, offering a formal quantification of its unpredictability, with higher values indicating more complex and less predictable dynamics [19]. According to Lau et al. [15], KSE is closely related to Les since it quantifies divergence with time between close values in the series. In a related vein, the Hurst exponent (HE) evaluates long-range temporal correlations, allowing the characterization of signals as persistent, anti-persistent, or uncorrelated depending on the degree of statistical dependence across time [20]. The most widely used estimator of HE is detrended fluctuation analysis (DFA), developed by Peng et al. [21], which assesses temporal correlations in non-stationary signals. The result of this measure is the exponent alpha, which can be used to identify if the signal is correlated (see Bryce and Sprague [22]).

In the context of neural signals, these measures have been widely used to investigate the dynamical properties of brain activity [5]. However, due to practical limitations when dealing with noisy and finite datasets such as EEG recordings, alternative metrics have gained prominence. In particular, fractal dimension indices provide robust estimates of signal complexity by quantifying self-similarity and irregularity. Among the most frequent are algorithms that estimate the fractal dimension directly from physiological time series. Among them, Higuchi’s fractal dimension (HFD) is probably the most extensively used. It estimates the length of the signal at different scales and calculates the fractal dimension as the linear regression of the resulting bivariate log(scale) and log(length) series [23]. Another widely used fractal dimension estimator is Katz’s fractal dimension (KFD), which provides an estimation based on Euclidean distances between samples in the signal and the diameter of the waveform (see Katz [24]).

### 2.2. Regularity

The most common regularity metrics are entropy measures, which quantify the rate of information production in a dynamical system [25]. These metrics include approximate and sample entropy (ApEn and SampEn) and multiscale entropy (MSE), among others. ApEn and SampEn measure the regularity of a signal by dividing it into epochs. The greater the frequency and similarity of these epochs, the lower the ApEn value and the more regular the signal [26]. MSE is obtained from computing SampEn on multiple temporal scales derived from the original signal and can capture complexity at different cortical rhythms [27]. Beyond sample-based measures, permutation entropy (PE) has become important in EEG analysis due to its computational efficiency and its ability to capture the temporal order of signal values. PE is particularly robust against outliers and high-amplitude artifacts, making it ideal for real-time applications and noisy datasets [28]. Furthermore, to address PE’s limitation in ignoring amplitude information, dispersion entropy (DisEn) has recently emerged as a powerful alternative. DisEn accounts for both the symbolic order and the amplitude fluctuations of the signal, providing a more stable estimate of complexity in short EEG segments [29].

Another regularity index which has been widely used in the study of EEG is the Lempel–Ziv complexity (LZC) [30]. LZC is a measure of the complexity of binary sequences that tests the randomness of the sequence by searching for repeated patterns in it. LZC is computationally less demanding than other complexity measures, is easy to implement, and can be applied to biological signals without any preprocessing steps [15]. Ibáñez-Molina et al. [31] developed the multiscale Lempel–Ziv complexity (MLZC), which is an extension of the classical Lempel–Ziv complexity, designed to analyze the complexity of a signal across multiple temporal scales rather than evaluating it only in its original form.

Notably, non-linear complexity metrics are not entirely independent of traditional spectral markers. Recent evidence suggests a strong relationship between signal entropy and the aperiodic component (1/f slope) of the power spectrum. Evidence suggests that both measures capture overlapping yet distinct aspects of neural dynamics; specifically, a flatter 1/f slope often correlates with increased entropy, reflecting a more desynchronized and information-rich brain state [32]. Interestingly, it has been shown that the application of the Haars wavelet transform is equivalent to the signal scaling process in MSE calculation [33]. This indicates a meeting point between classical signal decomposition and multiscale non-linear analysis. For a comparison of all of these reviewed measures, see Table 1 below.

## 3. EEG Complexity Across Cognitive Processes and States

### 3.1. Resting-State and Baseline Dynamics

Research has consistently demonstrated variations in EEG complexity not only between sleep and awake periods, but also across sleep stages [16]. Human sleep can be divided into two primary phases, the rapid eye movement (REM) phase and the non-REM phase, which are characterized by different physiological patterns, conscious content, sensory responsiveness, and muscle tone. REM sleep has been related to vivid dreaming, whereas non-REM sleep is commonly divided into three substages (N1, N2, and N3), from light to deep sleep, and is characterized by the deactivation of many brain areas active during wake and REM periods. Sleep phases alternate during the total sleep period, following well-known cycling patterns [34,35].

Since pioneering research from Achermann et al. [36] and Fell et al. [37], a number of studies employing different measures of complexity have systematically shown that EEG complexity is higher during wake and REM sleep and lower during non-REM sleep (see Ma et al. [16] for a review). In addition, complexity significantly diminished from shallow (N1) to deep sleep (N3). Complexity in REM sleep is higher than in non-REM sleep but lower than in wakefulness [38], although in some studies, the complexity of REM sleep is intermediate between that of N1 and N2 [39], and some others have found similar values of EEG complexity in REM and the N1 stage [40]. Similar trends in complexity modulation have also been reported in infants and newborns [16]. Duarte et al. [41] found that generalized weighted permutation entropy (GWPE) is good at differentiating sleep stages, particularly the transition between REM sleep and the N1 stage of non-REM sleep.

Mateos et al. [42] analyzed brain signals recorded via a scalp EEG, intracranial EEG (iEEG), and magnetoencephalography (MEG) in 27 subjects during wakefulness, different sleep stages, and epileptic seizures. They employed two complementary measures: permutation entropy (HPE), which captures the statistical unpredictability of the signal, and permutation Lempel–Ziv complexity (PLZC), which quantifies the deterministic information required to reconstruct the signal’s symbolic sequence. Wakefulness was consistently associated with the highest values of both HPE and PLZC, whereas epileptic seizures and deeper stages of slow-wave sleep showed marked reductions in both measures [42]. Notably, REM sleep exhibited complexity values close to those of the awake state, consistent with the cognitive activity associated with dreaming. These findings were robust across all three recording modalities (scalp EEG, iEEG, and MEG), underscoring the generalizability of non-linear approaches to indexing levels of consciousness. The authors interpreted these results within a framework in which optimal sensory processing requires variability in neural network configurations, reflected in higher signal complexity, whereas states of reduced awareness (seizures, deep sleep, and anesthesia) are characterized by more stereotypical, lower-complexity dynamics [42].

These variations in EEG complexity have also been found in other mammals. For example, González et al. [43] found that EEG complexity, measured with permutation entropy, was increased in rats during wake periods and reduced during sleep. This finding was independent of the electrodes’ cortical location.

However, research in humans has shown that changes in EEG complexity during sleep can vary across brain regions. Schartner et al. [44] studied global and local patterns of intracranial EEG during sleep and detected that the reduction in EEG complexity during non-REM sleep was evident at the global level and at the local level (groups of channels located in a single region), but EEG complexity was generally higher in the frontal lobe. More recently, Olejarczyk, Gotman, and Frauscher [45] calculated HFD from intracranial EEG data from 38 cortical regions. Consistent with previous research, HFD was higher during wakefulness and least complex during stage N3 of non-REM sleep. REM sleep was characterized by higher EEG complexity, similar to the awake state. However, there were important differences across regions. HFD was higher during wakefulness, especially in the frontal lobe, with two areas being particularly complex: the middle segment of the precentral gyrus and the inferior frontal gyrus. However, HDF did not change from wakefulness to stage N2 of non-REM sleep in temporo-occipital regions. In the transverse temporal gyrus, EEG complexity did not show any differences across sleep stages. Finally, the main difference between wakefulness and REM sleep was in the primary motor regions.

In general, these findings regarding EEG complexity during sleep suggest that brain activity becomes more coherent and ordered as the sleep stage goes deeper. However, variations in complexity can respond to different characteristics of wakefulness and sleep. On the one hand, low values of EEG complexity can reflect the reduction in cognitive control and attention that happens during sleep. However, this cannot explain the differences between REM and non-REM sleep, since in both stages, cognitive control is reduced. Another plausible explanation is that high complexity during wakefulness and REM sleep reflects the sensory richness characteristic of these states [15]. As we mentioned before, REM sleep has been associated with dreaming, which involves vivid phenomenological experiences. Research that involves awakening participants indicates that the cognitive content of non-REM sleep has lower sensory richness and contains more thoughts than perceptual experiences when compared to REM sleep.

In this regard, research has also explored EEG complexity related to the dreaming experience. With this goal in mind, Aamodt et al. [46] used a repeated awakening paradigm in sleep-deprived participants. They found that EEG complexity decreased with deeper stages of non-REM sleep, but they did not find differences in EEG complexity between dreaming and non-dreaming reports. However, they discovered a significant positive correlation between EEG complexity over the posterior cortex and thought-perceptual ratings of dream contents. However, this last finding has not been replicated. In a later study, the same authors [47] found no significant difference in EEG complexity between dream and non-dream awakenings, nor any significant relationship between complexity and subjective ratings of dream experiences within the same sleep stage (N2). Finally, Bajwa et al. [48] also employed an awakening paradigm but with healthy participants during propofol sedation. EEG complexity did not differ between dreaming and non-dreaming periods according to the participants’ reports.

Hou et al. [49] explored the association between pre-sleep EEG complexity and sleep quality and discovered that lower complexity before sleep was associated with decreased sleep latency. The authors suggested that low EEG complexity before going to sleep could have a facilitating role in the wake–sleep transition.

In conclusion, it seems that EEG complexity has been firmly demonstrated to be higher during wakefulness and reduces progressively from light to deep sleep. During REM sleep, EEG complexity is higher, more similar to wake periods or to the N1 stage of non-REM sleep. A feasible explanation could be that in deep sleep, fewer neurons are actively processing information, or that neurons are more synchronized and thus generate brain waves with less complexity. In REM sleep, the brain becomes highly active again. In this sense, cerebral blood flow and metabolism decrease in deep sleep, and remain about the same during REM sleep as in wakefulness [16]. Hence, we may highlight that a clear complexity gradient exists between NREM (low) and REM (high) sleep, probably mirroring the level of conscious experience.

### 3.2. Perception and Sensory Processing

Although the research on EEG complexity during sensory processing is not extensive, there are some interesting empirical findings. We will review the insights on primary sensory processing first, and then we will focus on multisensory integration.

In the visual domain, a study by Tong, Huang, Lang, and Chen [50] investigated the correlation dimension (D2) of the EEGs in a semantic categorization task (animal vs. non-animal image detection). The authors provided the D2 time evolution, showing a significant decrease in D2, peaking around 500 ms after stimulus presentation. This complexity reduction likely reflects a transient reduction of the dimensionality of cortical dynamics, associated with increased neural synchronization during stimulus processing. This interpretation is reinforced by the fact that the effect is more obvious in the parietal and occipital electrode locations. Moreover, the reduction peaks around 500 ms, a time window typically associated with higher-level perceptual categorization processes, which are the main goal of the task. In a different study, Branston, El Deredy, and McGlone [51] explored visual processing in an oddball task and found a similar decrease in complexity at 500 ms after image presentation. In addition, they found a decrease at around 300 ms. However, comparisons between these studies need to be cautious because Branston used the neural complexity (C_N_) measure introduced by Tononi, Sporns, and Edelman [52], which captures the properties of integration and the information of the system included in the signal, while D2 might be more sensitive to the effective dimensionality of the underlying dynamics of the signal, which might be more closely related to information. In another interesting study, Shourie, Firoozabadi, and Badie [53] investigated the EEG complexity of visual processing vs. the resting state in artist and non-artist participants. They found a decrease in values of ApEn in visual perception compared to the resting state; interestingly, artists showed higher ApEn in frontal sites, indicating more elaborate processing in this group. In this study, they also registered the ApEn in mental imagery. In this case, they found that visual processing yielded lower ApEn than mental imagery, confirming that visual perception is related to a decrease in complexity. A complementary investigation on simple visual repetitive stimulation has found that this effect tends to disappear with repetitions, indicating that cortical complexity is highly sensitive to habituation [54].

A different line of research in visual processing is the effect of stimulus complexity on EEG signals. The goal in this case is to study whether the complexity of stimulation is related to the complexity of cortical activity, as registered in the EEGs. Müller et al. [55] investigated whether participants visually exposed to chaotic vs. periodic pendulum movement exhibited different patterns of cortical function complexity. Interestingly, they found that the chaotic stimulus was associated with a higher pointwise dimension of EEG signals. More recently, Dorosti, Khosrowabadi, and Namazi [56] investigated HFD in EEGs from participants watching an animation of a fractal from low to high fractal dimensions. They found a direct correlation between both fractal dimensions in central and parietal regions of the scalp, showing that the increase in the complexity of the stimulus also led to more complex cortical processing.

Taken together, these two lines of research suggest that visual processing tends to decrease EEG complexity relative to the resting state, whereas greater stimulus complexity is associated with increased complexity in brain dynamics. While these findings may appear contradictory at first glance, they remain conceptually compatible. The reduction in complexity during visual perception may reflect a transient coordination of cortical activity towards sensory processing. In contrast, the increase in complexity observed for more complex stimuli may reflect the recruitment of additional dynamical degrees of freedom or neural processes required to represent more heterogeneous visual inputs.

Although there are fewer studies in the auditory domain, the trend in the pattern of results is quite similar. Xiang et al. [57] found a reduction relative to the baseline in the fuzzy entropy of the EEG when participants were presented with an auditory stimulus. They used a paired click sensory gating paradigm in a clinical context, although the non-clinical response was a reduction in complexity with stimulus presentation. Alnes et al. [58] also found a reduction of complexity measured with Lempel–Ziv complexity after auditory stimulation; interestingly, this effect was more evident during sleep stages than in wake states. This effect was attributed to the appearance of a large-amplitude k-component associated with stimulation during sleep (see [59]). However, Veyrié et al. [60] found that the awareness of auditory stimuli in a tone detection task yielded higher EEG entropy than subliminal perception (or unconscious listening). In this case, the effect trends in an opposite direction, although we need to consider that the critical comparison was not baseline vs. auditory processing, but rather consciously perceived vs. consciously missed. It could be possible that the conscious construction of the sensory input would require more information processing and integration, reflecting greater entropy in the physiological signals.

Regarding the effect of the manipulation of the complexity of auditory stimuli, Birbaumer et al. [61] presented participants with weak and strong chaotic music and discovered that strong and periodic music produced a higher D2 in the EEG. This experiment was designed in the context of the neural resonance hypothesis of music perception, in which music perception is explained not as a reaction but as an entrainment or coupling with the environment. In the same line of research, Carpentier et al. [62] discovered that the complexity of music was positively related to the complexity of the EEG. When the task, in addition to sensory processing, involved emotions and reward in participants, this effect decreased. This effect suggests that stimulus–brain complexity matching might mainly be a characteristic of low-level sensory processing. Overall, experiments in the auditory domain are well-aligned with those in the visual domain. However, this parallelism was not evident in a study by Orlowski and Bola [63]. They designed a study to directly compare EEG complexity in the visual and auditory modalities. They measured the LZC of the EEG while participants were presented with short video clips or segments extracted from audiobooks. The results indicated that visual stimuli, when compared with auditory stimuli, elicited higher LZC. In addition, they found that the LZC of resting EEGs was at an intermediate level between visual and auditory conditions. Hence, we should be cautious when trying to generalize complexity findings to different experimental designs.

To date, studies on the complexity of electrophysiological signals in other sensory modalities are scarce. In one experiment conducted by Tseng and Lo [64], evidence from a visuotactile paradigm suggests that complexity measures may capture the dynamic reorganization of cortical activity associated with somatosensory processing. Changes in multiscale entropy were reported in parietal locations during tasks manipulating hand proximity to visual stimuli. Finally, it is interesting to note that early work on animal sensory processing suggested that perception may involve transitions between complex spatiotemporal activity patterns in cortical networks. In particular, studies by Walter J. Freeman (e.g., [65]) in animal models showed that olfactory perception is associated with rapid shifts between distributed activity states that can be characterized in terms of rapid shifts between distributed activity states that can be described in terms of attractor dynamics. However, to our knowledge, comparable evidence in humans remains absent.

### 3.3. Attention, Executive Control, and Working Memory

Attention can be studied from diverse perspectives, from selective filtering to the simultaneous management of multiple information streams. These processes are supported by large-scale control systems, particularly frontoparietal networks. Within these networks, the prefrontal cortex (PFC) plays a central role by maintaining task goals and exerting top-down bias over distributed brain regions to guide behavior [66,67,68].

From a dynamical systems perspective, attentional states are associated with distinct patterns of neural complexity. Focused attention has been characterized by reduced entropy at specific temporal scales, reflecting a more constrained and stable neural regime. For instance, lower frontal MSE during inhibitory control tasks has been associated with higher accuracy and reduced reaction time variability, suggesting that efficient performance depends on the tightening of these dynamics [69,70,71]. This interpretation is consistent with the idea that variability in brain signals reflects the capacity to access a repertoire of functional states, which can be selectively constrained during goal-directed behavior.

Divided attention and sustained attention tasks require greater flexibility in network coordination. Under these conditions, the brain must balance integration and segregation across distributed systems, often operating in a metastable regime that allows rapid transitions between states [72]. Empirical evidence demonstrates that EEG complexity metrics effectively track these dynamics; specifically, frontal entropy measures (e.g., ApEn, SampEn, and FuzzyEn) and multiscale indices such as the Multiscale Sample Entropy Index (MSEI) and Multiscale Fuzzy Entropy Index (MFEI) have been shown to reliably differentiate levels of sustained attention and enable accurate classification of attentional states in continuous tasks [73]. Crucially, Wan et al. [73] demonstrated a direct positive correlation between attentional engagement and neural dynamics. They measured EEG while participants were performing a sustained attention task, and classified epochs with response times significantly shorter than a critical value as “high attention state”, epochs with response times significantly longer than a critical value as “low attention state”, and the rest as “medium attention state”. Results showed that higher attention states were associated with greater EEG complexity at frontal sites. This finding suggests that the cognitive effort required for high performance is associated with the richer and more complex coordination of frontal networks. Taking together all this evidence regarding attention, we may conclude that EEG complexity generally decreases during focused attention, reflecting a more constrained neural state, and might increase with augmented sustained attention.

Working memory (WM) supports the maintenance and manipulation of task-relevant information and is a core component of executive control. Early evidence using dimensional complexity measures, such as D2, showed that increasing WM load reduces the effective degrees of freedom in brain activity, thereby constraining the neural repertoire under high cognitive demands [74]. Importantly, this reduction was more pronounced in individuals with faster responses, highlighting the functional relevance of these dynamics. In addition, EEG decoding demonstrates that working memory load is encoded across distributed temporal, spatial, and frequency features, with cross-frequency coupling contributing to the coordination of neural activity across bands [75]. Together, these findings suggest that working memory involves the integration of information across multiple scales rather than being reflected in single-dimensional measures of neural activity.

Furthermore, alterations in these dynamics have been observed in both developmental and clinical contexts. For example, increases in EEG complexity, often quantified using entropy-based measures such as MSE or permutation entropy, across development have been associated with the expansion of the brain’s dynamic repertoire [76]. This developmental trajectory has been further characterized using multiscale entropy (MSE), showing that signal complexity increases significantly from childhood through adolescence and into adulthood at finer scales, whereas reduced complexity is related to coarse scales [77,78,79]. Regarding clinical contexts, decreased entropy and reduced signal variability have been linked to impaired cognitive control, as observed in attention-related disorders [71].

Cognitive flexibility, closely intertwined with working memory, is a central component of executive control, which is the ability to adapt behavior to changing goals. It is commonly studied using task-switching paradigms, in which individuals alternate between tasks requiring different stimulus–response mappings. A robust finding in this literature is the “switch cost”, reflected in slower responses and increased errors when switching tasks compared to repeating them, which is thought to index the cognitive control processes required for task-set reconfiguration [80,81]. From a neural perspective, task switching involves the dynamic updating of task representations, a process strongly associated with prefrontal control mechanisms [66]. These transitions require the brain to move flexibly between different functional states, a process that can be captured using measures of neural complexity. In task-switching paradigms, brain signal complexity increases as task difficulty rises, suggesting that the brain recruits additional resources to adapt to cognitive conflicts [82]. Specifically, Grundy et al. [82] observed that this increase in complexity—particularly at finer timescales—is associated with better performance when adaptively modulated, as individuals who showed a greater ability to “upregulate” their neural complexity during task conflicts exhibited lower behavioral costs and faster reaction times. Complementing this, a multi-parametric approach involving Lyapunov exponents, fractal dimensions, and SampEn has further characterized these dynamics under varying cognitive loads as complementary measures of neural dynamics. Parbat and Chakraborty [83] demonstrated that increasing mental workload is associated with changes in brain dynamical signatures, particularly within the alpha and beta frequency bands. These findings suggest that the brain maintains adaptive stability in information processing by dynamically diversifying its underlying complexity, effectively leveraging non-linear dynamics to respond to external demands. Furthermore, factors such as cardiorespiratory fitness have been shown to enhance executive functioning and EEG entropy, indicating improved efficiency in information processing [84].

Rather than constituting independent processes, attention, working memory, and cognitive flexibility can be understood as different expressions of a common mechanism, the dynamic reconfiguration of large-scale brain networks under varying task demands [85]. Within this framework, cognitive control depends on the brain’s ability to balance stability and flexibility, constraining neural dynamics during focused processing while maintaining sufficient complexity to allow rapid adaptation. Non-linear EEG metrics, such as entropy measures, including multiscale entropy and sample entropy, fractal dimension, Lempel–Ziv complexity, and correlation dimension, provide a unified framework to quantify these processes, capturing how the brain navigates its functional repertoire across cognitive states [86]. Together with theoretical models of prefrontal control [66], these approaches suggest that cognitive performance emerges from the dynamic regulation of neural complexity in response to task demands.

### 3.4. Complexity Markers of Encoding, Consolidation, and Retrieval in Long-Term Memory

Long-term memory (LTM) is one of the most widely studied phenomena in experimental psychology. Understanding how experiences are encoded, stored, and later retrieved has been a central objective of cognition research. Consequently, a wide range of behavioral paradigms and neurophysiological techniques have been developed to investigate the mechanisms underlying memory processes (e.g., [87]). In cognitive neuroscience in particular, considerable effort has been devoted to identifying the neural dynamics that support the different stages of memory processing, including encoding, consolidation, and retrieval. Within this context, EEG measures have been used to study the temporal dynamics and neural patterns of these processes (see [88]).

Research on LTM also includes measures of neural signal complexity derived from EEGs. In the context of complexity, the encoding, consolidation, and retrieval of memories can be understood as processes involving the reconfiguration of large-scale neural dynamics. Some studies have specifically examined how EEG complexity changes during different stages of memory processing. For example, entropy measures calculated during encoding have been shown to predict subsequent retrieval performance. This result suggests that richer or more differentiated neural dynamics during stimulus processing may facilitate the formation of durable memory traces [89]. In addition, Sheehan, Sreekumar, Inati, and Zaghloul [90] conducted an experiment in which the relationship between the SampEn of resting intracranial EEGs and successful memory was investigated. They used a paired-associates task in which participants needed to learn paired words for later recall using one of the word members as a cue. The results indicated that participants with higher intracranial EEG SampEn displayed better performance in the task and better recall. This effect was also found within participants, that is, when SampEn for different trials in single participants was related to recall. This experiment suggests that neural variability captured with SampEn is related to the cognitive flexibility needed for task performance. These results might be extended to the clinical context by considering the early work by Lutzenberger, Flor, and Birbaumer [91], where they found that different memories of painful events elicited higher EEG D2 in participants with chronic pain than in the healthy group. The increased dimensionality of EEG trajectories in chronic pain patients may reflect the recruitment of additional memory resources during recall. In addition, reduced complexity has been observed in conditions characterized by memory impairments, such as neurodegenerative disorders (e.g., [92]). Consequently, successful memory performance tends to be associated with more differentiated or complex neural dynamics.

Building on the link between successful recall and high complexity, one might hypothesize that forgetting is associated with reduced complexity. To the best of our knowledge, only one study addressed this question. Angsuwatanakul et al. [93] gave participants a visual memory task where they chose which images they wished to memorize and which to forget. In a later recognition test, as expected, the frontal regions of participants showed decreased complexity when measured with multiscale SampEn for the to-be-forgotten images.

### 3.5. Higher-Order Cognition: Intelligence, Creativity, and Problem-Solving

The study of higher-order cognition has undergone a significant paradigm shift, moving beyond traditional spectral power analysis toward characterizing brain dynamics as a complex system. Recent research has employed non-linear metrics—such as multiscale entropy, neural criticality, and latent state modeling—to capture the information richness and functional flexibility of the neural networks underlying fluid intelligence, creativity, and complex problem-solving.

#### 3.5.1. Creativity and Divergent Thinking

The neurophysiology of creativity is defined by the brain’s ability to transcend conventional patterns and generate original associations. Creative verbal processes are associated with a marked increase in EEG power in the beta and gamma bands, a pattern not observed in non-creative tasks even when they possess high subjective complexity [94]. Complementing the role of high-frequency oscillations, evidence suggests that creative ideation is also characterized by an increase in EEG alpha power. This alpha synchronization is thought to reflect a state of internally oriented attention, effectively shielding the brain from external sensory interference to facilitate the generation of original associations [95].

From a complexity perspective, higher individual creativity in healthy elderly populations has been linked to increased resting-state EEG complexity, particularly across long timescales—reflecting low-frequency dynamics—in central, parietal, and temporal regions [96]. Notably, no such relationship has been found with intelligence (WAIS-III), suggesting that creativity specifically benefits from richer and less predictable long-range networks at rest. This contrasts with the loss of complexity typically observed in aging and dementia, where these metrics decline [97].

Early research using correlation dimension D2 demonstrated that divergent thinking elicits a more complex neural regime than convergent thought. In a series of tasks requiring either divergent or convergent thought, Mölle et al. [98] found that EEG dimensional complexity was generally higher during divergent thinking than during convergent thinking. Crucially, within the divergent mode, participants with higher creative performance (more and better ideas) showed lower frontal dimensionality than low-performing individuals, suggesting that the most creative solutions may emerge from an intermediate level of neural complexity rather than from maximal dynamical richness. This finding anticipates the notion of an “optimal window” of complexity for creative cognition, where excessive rigidity and excessive instability are both suboptimal.

Furthermore, in young adults, the production of original verbal associations is marked by modest but consistent increases in frontal complexity at fine-to-medium scales (faster dynamics), although inter-individual differences in MSE remain highly stable as a “trait” across conditions [99]. These results align with frameworks conceptualizing temporal brain signal complexity as a marker of the richness of internal states involved in creative processes [100,101].

More recently, Qi and Liu [102] combined an alternative uses task with a metacontrol paradigm and showed that both higher metacontrol and higher creative performance were associated with increased EEG MSE at coarse timescales, particularly in temporal, parietal, and occipital regions, and across multiple functional networks, including the visual network. These findings suggest that creative thinking and metacontrol share a common neurodynamic substrate relying on long-duration, large-scale neural interactions.

#### 3.5.2. Intelligence

This line of inquiry dates back to early non-linear analyses; for instance, Lutzenberger et al. [103] found that individuals with higher IQ scores manifest greater dimensional complexity during resting states, particularly in parietal regions. Interestingly, they observed that this IQ-dependent gap diminishes during task performance, as less intelligent subjects exhibit a more pronounced increase in neural complexity to meet cognitive demands—a finding that aligns with modern theories of neural efficiency and state-dependent modulation.

Complementing these findings, Kaur et al. [104] used multiscale entropy during both a verbal divergent thinking task and an inhibition task to test whether brain signal complexity might provide a common neural substrate for creative thinking, intelligence, and cognitive control. They showed that MSE was a sensitive neural indicator of originality as well as inhibition, and that MSE measured during divergent thinking and inhibitory states was associated with fluency and originality at specific scalp sites, but not with fluid or crystallized intelligence. These results support the view that neural complexity links creative thinking more tightly to cognitive control processes than to psychometric intelligence per se.

Fluid intelligence also relies on spatiotemporal complexity, but with a distinct profile. Using multivariate MSE (mMSE), Dreszer et al. [105] demonstrated that intelligence correlated with higher complexity at coarser timescales, particularly within the frontoparietal and temporoparietal regions. Notably, these patterns were modulated by sex: the relationship was more pronounced in parietal and occipital regions in men, while it predominated in frontal areas in women. This suggests that intelligence may rely on a dynamic organization that maximizes long-range integration while avoiding potentially noisy local variability, consistent with the global efficiency of small-world networks [106,107]. This is further supported by Thiele et al. [108], who demonstrated that MSE at coarse timescales—reflecting long-range neural interactions—is the most robust predictor of fluid intelligence. Interestingly, they also noted that intelligence may be associated with reduced complexity at finer scales, suggesting efficient neural organization that minimizes local noise while maximizing the diversity of global functional states.

#### 3.5.3. Problem-Solving

Beyond executive control, classic studies on problem-solving using D2 measures have demonstrated that convergent and static modes of thinking are associated with reductions in complexity, particularly in right parietal regions, whereas functional, operation-oriented modes maintain a higher number of active neural assemblies [109].

Finally, research in clinical populations highlights the importance of the modulation of complexity between resting and task states. In adults with ADHD, the transition from a complex resting state to lower frontal complexity during a go/no-go task is associated with better performance in control groups, whereas participants with ADHD exhibit reduced or inverse modulation [71]. Analogous results in other conditions suggest that both excessively low complexity and overly high, non-modulable complexity can be maladaptive [110].

Taken together, these findings suggest that EEG complexity metrics effectively capture the critical network dynamics underlying higher-order cognition. The “optimal” level of complexity is contingent upon contextual demands: tasks necessitating creative exploration may require higher neural complexity, whereas those demanding stringent inhibitory control appear to necessitate a localized reduction in complexity to maintain stability.

### 3.6. Mind-Wandering and Spontaneous Thought: Complexity Across Timescales

Recent research has begun to study mental states that are not directly related to external stimuli or to the ongoing task. These internally guided experiences have been labelled with several terms, including stimulus-independent thought, internally directed cognition, or mind-wandering (MW), among others. Mind-wandering is a frequent experience (it covers between 30% and 50% of waking brain activity), in which attention disengages from the ongoing external context and focuses on thoughts, mental images, or memories. This type of cognition may appear during the performance of an external task or when an individual is not engaged in any external task [111].

Linear measures of EEG have been extensively explored in mind-wandering. Evidence has consistently shown a reduction in the amplitude of diverse ERP components (P1, N2, and P3) during mind-wandering compared to on-task states. As these ERPs signal early sensory and cognitive responses to stimuli, their attenuation has been interpreted as evidence supporting the decoupling from the external environment during mind-wandering. Results involving spectral markers are more contradictory, but they generally converge on greater activity in the low-frequency range of delta, theta, and alpha, and a decrease in beta band activity during mind-wandering (see [3] for a review).

Research exploring non-linear EEG measures in mind-wandering is quite scarce. In a pioneering study, Natarajan et al. [112] studied different non-linear EEG measures (the correlation dimension, largest Lyapunov exponent, Hurst exponent, and approximate entropy) in participants during different mental states: under the normal resting state, listening to music, or with foot reflexology stimulation. Although they did not explicitly explore mind-wandering, this study is of interest because we can suppose that the participants in the resting state are mind-wandering, since there are no external stimuli. The results showed that all complexity measures were reduced under the influence of music and reflexological stimulation. Years later, Ibáñez-Molina and Iglesias-Parro [113] carried out the first study that addressed EEG complexity during mind-wandering. They generated a novel experimental paradigm, based on binocular rivalry, to study EEGs during different conscious experiences. They presented participants with a video that did not have matching visual and auditory inputs, and, using sample probes, asked participants at various moments whether they were responding to auditory stimuli, to visual stimuli, or were mind-wandering. They found that the fractal dimension of the EEG was higher when individuals were mind-wandering than when they were responding to visual or auditory stimuli, and this difference was extensively distributed over the scalp. Cnudde et al. [114] replicated and extended these results, exploring whether effects differed across timescales. They recorded participants’ EEGs while they completed a perceptual global and local task and asked participants with sample probes whether they were focused on the task or were mind-wandering. They found that EEG complexity, measured with multiscale entropy, decreased at medium timescales in centro-parietal regions and increased at coarse timescales in anterior and posterior regions during mind-wandering, as compared to the on-task state. Complexity at finer timescales indicates processing flexibility at local regions, whereas complexity at coarser timescales indicates processing flexibility across distal regions. Hence, the authors interpreted that, during mind-wandering, there was greater flexibility across distal brain regions, suggesting a higher capacity to switch between possible functional configurations.

Greater values of EEG complexity during mind-wandering have also been validated using neurocomputational models [31]. These authors simulated the oscillatory activity of the entire brain and explored the interaction between two main brain networks: the default mode network, usually associated with resting states, and the salience network, normally associated with the processing of external events. The model was fitted to a dynamical state, in which there was a transition from a global synchronized state to a metastable regime in which synchrony changed quasi-periodically. The fractal dimension of the simulated fitted EEG data was higher when the coherence in the default mode network was transiently increased vs. the salience network. This effect parallels the empirical data comparing mind-wandering and externally focused attention, respectively.

However, these findings can be modulated by the compared on-task state. Lu and Rodriguez-Larios [115] found opposing results when they compared EEG complexity in mind-wandering and breath focus states. They discovered that EEG complexity (measured with Higuchi’s fractal dimension, Lempel–Ziv complexity, and sample entropy) was reduced during mind-wandering relative to breath focus states. These apparently contradictory results can be understood if we consider the definition of mind-wandering. As we mentioned before, mind-wandering states are defined as those that are independent of external stimuli and of the ongoing task. However, in the research by Lu and Rodriguez-Larios [115], mind-wandering was compared to breath focus states, in which attention is also decoupled from external stimuli and internally directed. Then, it is possible that EEG complexity is high in internally directed cognitive states (higher when attention is directed by a task and lower when attention is internal, unrelated to the task at hand) and lower when cognitive states are directed to external stimuli.

Finally, Krile et al. [116] studied the relationship between mind-wandering, EEG complexity, and posterior learning. Consistent with previous research, they found that more frequent mind-wandering was associated with higher brain complexity across widespread timescales and electrodes. Interestingly, this increase in EEG complexity was associated with improved performance on a perceptual task following training. The authors suggested that increased complexity associated with mind-wandering enables neural flexibility, and this flexibility facilitates learning. Thus, patterns of EEG complexity associated with mind-wandering before task training may support subsequent learning by increasing flexibility to support the engagement of diverse brain networks and the generation of new behaviors.

### 3.7. Meditation and Altered States of Consciousness

Altered states of consciousness have been mainly studied with the administration of psychotropic drugs, as well as for exploring meditative states of consciousness. Regarding the first line of research, Li and Mashour [117] calculated the Lempel–Ziv complexity of spontaneous high-density EEGs (128 channels) in 15 healthy volunteers who were administered subanesthetic and anesthetic doses of ketamine. The key findings demonstrated clear dose-dependent effects. Subanesthetic ketamine was associated with elevated spatiotemporal LZC relative to baseline, consistent with the psychedelic phenomenology of enhanced subjective experiences [117]. During the anesthetic period, complexity alternated between low values (delta-dominant state S3) and high values (gamma-dominant state S4), reflecting the gamma burst pattern characteristic of ketamine anesthesia. Before recovery of consciousness, complexity stabilized at a level comparable to baseline. Li and Mashour [117] proposed that what is commonly referred to as “ketamine anesthesia” is in fact a fragmented state, rapidly alternating between anesthetic-like epochs (low LZC) and consciousness-like epochs (high LZC), consistent with the known dissociative properties of ketamine that preserve vivid disconnected consciousness (e.g., dream-like states) while disrupting connected environmental awareness.

With respect to meditation, several studies have explored EEG complexity in meditative states. Young et al. [118] examined EEG in 28 highly skilled meditators practicing six different techniques (shamatha, vipassana, zazen, dzogchen, tonglen, and deity visualization). EEG was recorded with a 16-channel system during both meditation and a mind-wandering control task, and non-linear dynamics were quantified using Lempel–Ziv complexity. The analysis revealed a significant main effect of state, with LZC scores consistently lower during meditation than during mind-wandering [118]. This reduction in complexity was observed across all six practices, suggesting that meditation, irrespective of technique, constrains the repertoire of neural configurations. In contrast, no significant differences emerged in power spectra across delta, theta, alpha, beta1, beta2, or beta3 bands, with only moderate effect sizes for alpha and beta1. The authors attributed this null spectral finding to the heterogeneity of practices, which may modulate different frequency bands in opposing directions and thus cancel out at the aggregate level [118].

Young et al. [118] argued that reduced complexity during meditation parallels the entropy reductions reported in other constrained states of consciousness, such as anesthesia and deep sleep. Directing attention toward specific sensory or cognitive targets that are shared features of the practices studied would decrease the number of accessible neural configurations and thereby reduce entropy. This work illustrates that non-linear measures such as LZC can detect meditation effects that are not apparent in conventional spectral analyses.

Aguerre et al. [119] extended the study of meditation-related brain dynamics to the trait level by investigating dispositional mindfulness in 120 meditation-naïve adults. Participants completed the Five Facet Mindfulness Questionnaire (FFMQ) and underwent 64-channel EEG recordings during a 5 min eyes-closed resting state and a 5 min learning task. The authors combined quantitative EEG (q-EEG) measures across theta, alpha, beta, and low-gamma bands with sample entropy as a non-linear index of signal irregularity. Higher FFMQ scores were significantly associated with reduced frontal gamma power at rest and lower global q-EEG power at rest, but not during task performance [119]. A regression model identified global resting-state power as the best predictor of dispositional mindfulness, explaining about 5% of the variance. With respect to complexity, SampEn during the learning task showed a marginally significant positive association with mindfulness scores, suggesting a trend toward higher entropy in more mindful individuals during active information processing [119]. These results were interpreted as evidence that more mindful individuals exhibit a “quieter” brain at rest—with reduced default mode network engagement and less spontaneous mind-wandering—alongside richer, more variable processing during task performance. This pattern parallels findings in expert meditators and supports the idea that non-linear EEG signatures linked to meditation expertise can also characterize naturally occurring individual differences in mindfulness, even in the absence of formal practice [119].

Walter and Hinterberger [120] offered a more mechanistic view of meditation-related dynamics by combining complexity and criticality analyses with traditional spectral measures. In 30 experienced meditators (average ≈ 20 years of practice), 64-channel EEGs were recorded during resting states (eyes open/closed), a reading task, and three meditation conditions: presence monitoring, thoughtless emptiness, and focused attention. The authors computed multiscale entropy, Higuchi’s fractal dimension, detrended fluctuation analysis (DFA) exponents, and neuronal avalanche statistics, alongside power spectral density. Compared with resting with eyes closed, both thoughtless emptiness and focused attention showed higher MSE and HFD, indicating increased neural complexity [109]. Focused attention produced particularly strong effects in MSE and was accompanied by increased gamma-band power. In all three meditation conditions, DFA exponents were reduced relative to rest, suggesting attenuated long-range temporal correlations and a shift toward more subcritical dynamics. The critical scaling exponent was lowest during focused attention and reading, linking heightened attentional demands to deviations from criticality [120]. Using partial least squares regression followed by ROC analysis, Walter and Hinterberger [120] showed that sample entropy achieved the highest discrimination accuracy among non-linear measures, followed by HFD and DFA, while gamma-band power and global spectral power were the most informative linear features. The negative correlations observed between the critical exponent and complexity indices caution against the simplistic assumption that higher complexity always implies operation closer to a critical point. Instead, their results suggest that meditation can increase local signal complexity while simultaneously shifting large-scale dynamics away from criticality [120].

Taken together, these studies converge on several conclusions (see the Table 2 for a summary). First, non-linear complexity measures are highly sensitive to meditation-induced changes in brain dynamics, even when traditional spectral analysis fails to detect significant differences (e.g., [118,120]). Second, the direction of complexity changes during meditation is not unequivocal; it depends on the specific metric, the baseline comparison, and the meditation category. When compared to active mind-wandering, meditation tends to reduce complexity (as indexed by LZC), suggesting a more constrained neural repertoire [118]. However, when compared to passive resting with eyes closed, certain meditation states—particularly focused attention and thoughtless emptiness—appear to increase complexity as captured by entropy and fractal measures [120]. Third, increased entropy has been consistently associated with psychedelic-induced expanded states of consciousness (e.g., under ketamine [117]) and with wakefulness relative to unconscious states [42]. Nonetheless, meditation, which often aims to reduce the contents of consciousness, may produce either increases or decreases in complexity depending on the practice and the dimension of consciousness that is being modulated. The ketamine data illustrate this duality particularly well: subanesthetic doses that induce psychedelic phenomenology elevate complexity above the baseline, whereas the anesthetic dose produces a fragmented state that alternates between consciousness-like and anesthesia-like complexity levels [117]. Fourth, the relationship between criticality and complexity warrants careful interpretation. Walter and Hinterberger [120] demonstrated that the critical scaling exponent was negatively correlated with entropy and fractal dimension measures, cautioning against the common assumption that higher complexity necessarily implies dynamics closer to the critical point. This distinction has direct theoretical implications for theories such as integrated information theory and the entropic brain hypothesis, which sometimes conflate these concepts. Finally, the inclusion of dispositional mindfulness as a correlate of neural complexity [119] opens a complementary avenue for understanding how individual differences in attentional regulation—without formal meditation practice—are reflected in the non-linear dynamics of resting-state EEG. The convergence between the patterns observed in expert meditators and those found in naturally mindful individuals strengthens the ecological validity of complexity-based EEG metrics as indices of consciousness-related traits and states.

## 4. Discussion

**Figure 1:**
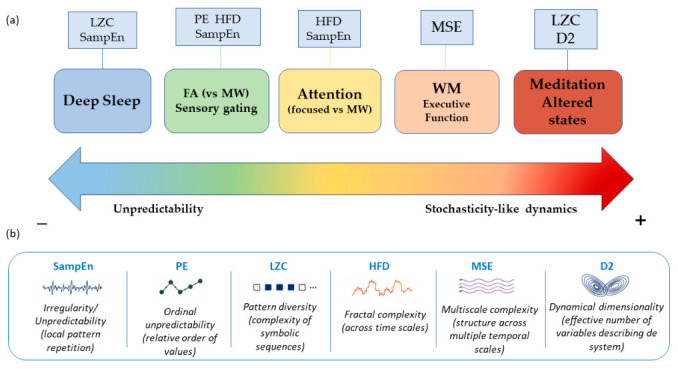
Conceptual framework of the predictability–regularity continuum in neural dynamics and cognitive processing. (**a**) Cognitive domains and states commonly investigated using non-linear EEG measures are arranged along a conceptual continuum ranging from more irregular and unpredictable neural dynamics (**left**) to increasingly stochastic-like dynamics (**right**). The non-linear metrics displayed above each domain correspond to the measures most frequently associated with these processes in the literature reviewed in this manuscript. (**b**) Summary of the principal aspect of signal complexity captured by each non-linear metric. These measures quantify complementary properties of neural signal complexity rather than a single underlying construct.

In this review, we have summarized the current evidence on the relationship between cognitive processing and EEG complexity across a wide range of functions and states, including resting wakefulness and sleep, perception and sensory processing, attention and executive control, long-term memory, and higher-order cognition. The findings reviewed across these different cognitive domains suggest a common organizational principle in brain dynamics, as synthesized in our conceptual framework (see Figure 1). Rather than a monolithic increase or decrease in complexity, cognitive processing appears to rely on a flexible transition along a predictability–regularity continuum. Domains requiring high internal exploration and information integration, such as working memory and creative thinking, consistently exhibit an expanded dynamic repertoire, reflected in higher complexity values. Conversely, states characterized by metabolic conservation or highly focused sensory filtering, such as deep sleep or early sensory gating, involve a strategic shift toward more predictable and redundant dynamics.

Although the literature reviewed here offers the valuable insights indicated above, the findings across different cognitive domains are not always consistent. It would be possible to divide these discrepancies into three main sources of divergence: (i) the specific aspect of complexity measured, as metrics of temporal predictability may yield different results than metrics of pattern regularity; (ii) variations in experimental task design; and (iii) the lack of standardized resting-state baselines, given that the physiological “starting point” of complexity varies considerably across studies, directly affecting the interpretation of task-related shifts.

Acknowledging this variability across studies, they support the idea that neural complexity metrics capture meaningful aspects of large-scale brain dynamics that might not be accessible through traditional approaches such as ERPs or band-limited spectral power [7,15,121,122,123]. Rather than providing a single, unitary index, complexity measures appear to reflect a family of complementary properties—predictability, regularity, multiscale temporal structure, and pattern diversity—that jointly characterize how the brain flexibly reconfigures its functional repertoire in response to internal and external demands [15,23,27,30,108,111].

It is important to note that EEG complexity has been linked to the coordinated activity of distributed neural assemblies. Building on Hebb’s notion of cell assemblies as functional units of cortical processing [124], non-linear EEG analyses interpret increases in complexity as reflecting a richer repertoire of simultaneously active or rapidly reconfiguring ensembles, rather than mere amplitude changes in traditional frequency bands [91,98,113,125]. Oscillatory activity and its interactions further enrich the signal’s temporal structure. Fast rhythms (beta and gamma) allow for very detailed local encoding, while slower rhythms (delta, theta, and alpha) define broader temporal integration windows, and coupling between frequencies organizes information across these scales [126,127,128,129,130]. In this context, desynchronization of dominant rhythms can increase complexity by allowing multiple ensembles to co-activate and generate more diverse configurations, while strong synchrony in slow bands can locally reduce complexity while supporting functions such as attentional selection or memory consolidation [38,98,131,132]. Temporal hierarchies and coupling between frequencies naturally give rise to multiscale correlations that appear in multiscale entropy profiles, thus linking oscillatory organization with measures of neural complexity [7,27,108]. In addition, we also note that these metrics are not mere indicators of the noise present in the signal; it would be better to think about the majority of complexity measures as indicators of cortical differentiation that might reflect a diversity of processes interacting in coordination [52,85]. Particular measures may show a different aspect of this diversity, as we stated in Section 1.

Starting with the domain of sleep, one of the most robust findings across studies concerns the modulation of EEG complexity by global states from wakefulness to deep sleep, as well as pathological states, including epileptic seizures or anesthesia. As we reviewed above, numerous studies have shown, in general, higher complexity during wakefulness and REM sleep and lower complexity during non-REM sleep, with a progressive decrease from N1 to N3 [16,38,39,40]. Functionally, these results may support frameworks in which higher complexity reflects a richer repertoire of network configurations during awake states, and hence, can be associated with sensory richness and cognitive engagement [15,133,134]. Deep non-REM sleep and seizures would correspond to more stereotyped and synchronized dynamics with reduced variability, whereas wakefulness and REM sleep maintain a multiscale organization compatible with ongoing perception and thought [16,42]. However, studies comparing dreaming versus non-dreaming reports within the same sleep stage show mixed results [46,47,48], suggesting that the relationship between complexity and conscious experience is not one-to-one and may depend on specific content, such as sensory richness, self-referential mentation, or temporal continuity.

When considering specific brain states during wakefulness, it is essential to account for a wide variety of cognitive processes that could modulate the complexity of EEG metrics. In the sensory domain, evidence shows a general pattern, characterized by a decrease in complexity (both entropy and dimension) from resting to sensory processing (e.g., [51]). These results suggest that the arrival of a salient stimulus may transiently constrain neural dynamics into a more ordered, low-dimensional regime, reducing complexity relative to the resting state [50,57,58]. At the same time, manipulations of stimulus complexity show that more complex inputs can increase EEG complexity [55,61,62]. The fact that stimulus–brain complexity matching is evident in low-level sensory processing [61,62] may indicate a correspondence between the richness of network configurations in primary sensory regions and the richness of the stimulation. It would be reasonable to consider that low-level processing would need more segregated processors to capture the richness of complex stimuli than in the case of less complex ones.

Studies on attention, executive control, and working memory converge on the idea that optimal performance depends on the ability to flexibly modulate complexity rather than on uniformly high or low values. Focused attention and inhibitory control are often characterized by reduced frontal MSE or entropy at specific temporal scales, which are associated with higher accuracy and less reaction time variability [69,70,71]. This pattern fits theoretical models of prefrontal control, in which top-down bias from the PFC stabilizes task-relevant representations and constrains variability within frontoparietal networks [66,67,68]. In other words, EEG complexity can be downregulated by top-down processing via a processing bias toward a given task goal. Conversely, divided attention, sustained monitoring, and task-switching paradigms often show increases in complexity when task difficulty or conflict rises. For example, Grundy et al. [82] found that higher frontal complexity under conflict was linked to lower switch costs and faster reaction times, indicating that individuals who can “upregulate” complexity under demanding conditions perform better.

Working memory studies, in general, show that complexity in WM performance depends on the memory load and accuracy in the responses of participants [75,132]. One pattern that might be extracted from the reviewed work is that accuracy in the responses of participants is often associated with higher neural complexity [132,135]. However, it is important to note that behavioral measures such as accuracy may not always serve as a fully reliable indicator for the underlying intensity of cognitive loads [75]. Although there is not a clear pattern of results in attention, control, and WM, the evidence indicates that focusing on one single processing task is related to low complexity, low cortical diversity, and high predictability, and divided attention and control resources to optimize WM lead to a high level of complexity.

Regarding long-term memory, studies provide converging evidence that richer and more differentiated neural dynamics support successful encoding and retrieval. Entropy-based measures during encoding have been shown to predict later recall, suggesting that more variable and structured dynamics facilitate the formation of durable memory traces [89,90]. The few studies on intentional forgetting suggest a complementary pattern. Angsuwatanakul et al. [93] observed decreased frontal MSE for to-be-forgotten images, consistent with a relative simplification of neural dynamics during suppression. This asymmetry between successful encoding/retrieval (higher complexity) and forgetting (lower complexity) might indicate that memory and encoding, as constructive processes, require a large variety of different processors, while forgetting, a more focused function, would act as simple processing towards the deletion of information.

In higher-order cognition, it has been shown that high and moderate levels of complexity are related to high task achievement. For example, creative performance has been linked to increased resting-state complexity at long timescales [96] and to task-related modulations of dimensional complexity and MSE during divergent thinking, often pointing to an optimal intermediate level rather than maximal complexity [94,95,98,99,100,101,102,104,109]. For intelligence, multivariate MSE and related approaches show that fluid intelligence is preferentially associated with higher complexity at coarse scales in frontoparietal and temporoparietal networks [103,105,106,107,108]. Classic problem-solving studies and recent clinical research in ADHD highlight that both overly low and rigid complexity and excessively high, non-modulable complexity can be maladaptive [71,109,110]. Therefore, in the case of many higher-order cognitive processes, exhibiting high-to-moderate EEG complexity is likely associated with a richer repertoire of neural assemblies or cortical diversity.

In sum, we believe this review has highlighted some general consistent patterns (although with logical inconsistencies that can be related to different measures and experimental paradigms). These patterns are congruent with the view of neural complexity as reflecting neural functional richness or the diversity of neural repertoires. If we consider global states, it seems clear that reduced consciousness states (sleep, anesthetized states, etc.) are characterized by low neural complexity, while expanded consciousness states (for example, psychedelic-induced states) are related to higher levels of EEG complexity. When we consider not global states but specific cognitive functions, the pattern is more complex. In sensory processing and focused attention, EEG complexity is usually lower than in resting-state and mind-wandering states. However, in all cognitive functions, there seems to be a direct relationship between task performance and neural complexity, and also between the cognitive demands of the task and neural complexity. Thus, the optimal level of brain complexity seems to depend on the task requirements. A lower complexity may represent a reduced need for flexibility in more predictable tasks, in which focused or selective processing is needed [116]. On the contrary, higher EEG complexity would reflect a greater flexibility to permit quicker transitions between potential network configurations, resulting in better learning, divergent thinking, and improved performance. From an information-theoretic perspective, this “optimal complexity” could be formalized as a dynamic optimization problem: the brain must maximize its information capacity (entropy) to explore different network configurations while remaining within structural and functional constraints (predictability) to prevent a collapse into chaotic randomness. This balance directly aligns with computational frameworks of neural metastability, where optimal cognitive performance arises when the system maintains a wide dynamic repertoire without becoming trapped in rigid synchronization or unstructured noise.

## 5. Methodological Considerations and Future Directions

Although the accumulated evidence shows that EEG complexity metrics are powerful tools to study brain–cognition relationships, several methodological challenges remain. Different measures—correlation dimension, Lyapunov exponents, entropy-based indices, fractal dimensions, LZC, and multiscale variants—are sensitive to distinct aspects of the signal and do not necessarily yield convergent results within the same dataset [15,17,18,19,20,23,24,27,30]. This heterogeneity complicates cross-study comparisons, highlighting the urgent need for systematic evaluations of metric properties, including their robustness to preprocessing, recording conditions, and noise.

A second issue is the intrinsic scale dependence of complexity in both time and space. While multiscale entropy and multiscale Lempel–Ziv complexity begin to address temporal aspects [27,136], many studies still rely on univariate, single-scale analyses, and fewer explicitly relate specific scales to defined cognitive operations or to the balance between functional integration and segregation [7,52,85,108,137,138,139]. Third, experimental designs often focus on static contrasts, while complexity metrics are particularly well-suited to capture dynamic transitions and metastable regimes, as illustrated by work on EEG microstates, neural avalanches, and criticality [8,9,140,141,142,143,144,145].

Finally, complexity must be integrated with other aspects of neural dynamics, especially the oscillatory structure and the aperiodic 1/f component. Changes in complexity frequently co-occur with shifts in spectral exponents, oscillatory power, and coupling, which together reflect excitation–inhibition balance and network topology [38,126,127,128,129,130]. Recent work shows that states of high arousal or cognitive demand often present flatter aperiodic slopes and higher complexity, while deep sleep and anesthesia combine steeper slopes with reduced complexity [44,108,117,146]. At the same time, correlations between complexity and critical exponents are not always positive, warning against simple equations between “more complex” and “closer to criticality” [120].

EEG complexity should not be conceived as a single, monolithic marker, but as a multidimensional construct that captures how the brain balances order and variability across temporal and spatial scales [52,85,111,114]. The studies reviewed indicate that this balance is systematically modulated by global brain states, sensory input, attentional demands, memory processes, spontaneous thought, meditation, and higher-order cognition, and that deviations from an optimal window are associated with cognitive impairments and clinical conditions [110,115,116,119,122,134]. Integrating non-linear dynamics with classical electrophysiological and network approaches may therefore be a promising path toward a more comprehensive understanding of how complex neural activity gives rise to cognition and consciousness.

Additionally, we would like to point out that a critical challenge for the field is the lack of standardization in metric parameters (e.g., embedding dimensions or threshold values). Future research should prioritize the use of public benchmark datasets to validate how different complexity indices respond to standardized cognitive loads. Moreover, as suggested by the recent literature, the impact of preprocessing pipelines—such as filtering bands and artifact rejection methods—must be systematically evaluated, as they can significantly bias non-linear estimates [147]. Adopting multivariate and spatially distributed approaches will be crucial for capturing how complexity emerges from the interaction between distant cortical hubs rather than isolated channels.

Finally, an intriguing and highly relevant future avenue for applied neurotechnology lies in the intersection of non-linear dynamics and modern machine learning (ML). While deep learning models excel at decoding EEG signals for brain–computer interfaces (BCIs), they remain vulnerable to cross-subject variability and malicious data manipulation. Future work should explore whether handcrafted, non-linear complexity metrics can serve as invariant, robust features to enhance ML pipelines. Specifically, integrating these features could offer a complementary layer of defense within frameworks such as alignment-based adversarial training (ABAT) [148] or general adversarial robustness paradigms [149], bridging the gap between theoretical neurophysiology and robust neural engineering.

## Figures and Tables

**Table 1:** Comparison of metrics by category. Features, methodological considerations, advantages, and limitations are described.

Metric	Category	Main Feature Measured	Methodological Considerations	Advantages/Limitations
Correlation Dimension (D2)	Predictability	Effective dimensionality: independent variables to describe the system’s dynamics.	Requires long recordings, stationarity, and embedding parameter selection.	Highly sensitive to system structure, but requires long, stationary signals.
Lyapunov Exponents (Les)	Predictability	Rate of divergence of nearby trajectories; sensitive to initial conditions (chaos).	Requires phase-space reconstruction and relatively long, low-noise signals.	Excellent for identifying chaotic behavior, but extremely sensitive to noise in biological signals.
Entropy (SampEn, FuzzyEn)	Regularity	Signal regularity and information production rate; probability of pattern repetition.	Depends on parameter selection (m, r); suitable for relatively short EEG segments.	Robust for short EEG time series. FuzzyEn is less sensitive to parameter selection (m, r) than SampEn.
Permutation Entropy (PE)	Regularity	Measures information by estimating relative order of consecutive values in the time series.	Depends on embedding dimension and delay; no amplitude discretization required.	Efficient calculation and noise resistance.
Dispersion Entropy (DisEn)	Regularity	Diversity of patterns considering both amplitude and temporal order.	Requires discretization into classes and selection of symbolization parameters.	More stable than PE for short signals and less sensitive to noise than SampEn.
Fractal Dimension (HFD)	Fractality	Self-similarity and signal irregularity across different timescales.	Depends on scale parameter selection and preprocessing procedures.	Computationally efficient and highly sensitive to subtle changes in cortical dynamics.
Lempel–Ziv Complexity (LZC)	Regularity	Diversity of patterns and randomness in symbolic (binary) sequences.	Requires signal discretization or binarization.	Does not require complex preprocessing; ideal for distinguishing levels of consciousness/arousal.
Multiscale Measures: Entropy (MSE) and Lempel–Ziv (MLZC)	Multiscale	Structural complexity across multiple timescales (e.g., nesting of cortical rhythms).	Require longer recordings and involve scale selection procedures.	Captures info that single-scale metrics miss; distinguishes between white noise and true biological complexity.

**Table 2:** Summary of EEG complexity shifts and functional significance across cognitive functions.

Cognitive Domain	Main Complexity Shift	Metrics	Key Functional Significance
Perception and Gating	Decrease during stimulus processing	LZC, PE, SampEn	Transition to a more predictable and constrained state for efficient sensory filtering.
Attention	Decrease during focused/external attention	MSE (fine scales), DFA, LZC	Neural specialization and reduction of “background noise” to prioritize task-relevant info.
Working Memory	Increase with cognitive load	MSE (broad scales), LZC, ApEn	Recruitment of a large dynamic repertoire to maintain and manipulate information
Higher-Order Cognition	Increase in creativity/intelligence	MSE, HFD, LZ	“Neural readiness” and flexibility to explore different functional network configurations.
Meditation	Variable (depends on technique)	SampEn, MSE, LZC	Focused Attention: Decrease (regularity). Open Monitoring: Increase (diversity of thought).
Sleep	Gradient: REM (High) > NREM (Low)	LZC, MSE, PE	Directly correlates with the level of conscious experience and thalamocortical integration.

## Data Availability

No new data were created or analyzed in this study. Data sharing is not applicable to this article.
